# Structural basis for transport and inhibition of nucleotide sugar transport in pathogenic fungi

**DOI:** 10.21203/rs.3.rs-7213965/v1

**Published:** 2025-08-05

**Authors:** Joanne L. Parker, Justin C. Deme, Bjarne Feddersen, Susan M. Lea, Simon Newstead

**Affiliations:** 1 Department of Biochemistry, University of Oxford, Oxford, OX1 3QU, UK;; 2 The Kavli Institute for Nanoscience Discovery, University of Oxford, Oxford, OX1 3QU, UK,; 3 Center for Structural Biology, Center for Cancer Research, National Cancer Institute, Frederick, MD 21702, USA.

## Abstract

GDP-Mannose transporters are Golgi-localised solute carriers that are essential for the virulence of pathogenic fungi, serving as critical components of fungal glycosylation pathways. However, the mechanism by which nucleotide sugars are recognised and transported across the Golgi membrane remains unclear, hindering efforts to develop effective inhibitors that could serve as novel antifungal agents. Here, we present cryo-EM structures of the GDP-Mannose transporter, Vrg4, from *Candida albicans* in complex with nanobodies and in both the cytoplasmic and Golgi-facing states. Structural comparisons between these two states, in addition to a GDP-mannose bound structure, demonstrate the importance of ligand movement during transport. Additionally, we demonstrate the ability of the nanobodies to specifically inhibit Vrg4, presenting proof-of-principle that nanobodies can be used as effective inhibitors of nucleotide sugar transport and glycosylation in cells.

## Introduction.

Invasive fungal pathogens have become a significant concern in healthcare systems worldwide. Fungal skin infections affect one in three people and cause over a million deaths each year. Candidiasis, caused by the overgrowth of mainly *Candida albicans*, accounts for approximately 70% of fungal infections^1,2^. A particular concern is the rising mortality rate among immunocompromised patients with Candidiasis, which is now nearly 40%^1^. For invasive fungal infections, only four major classes of antifungal agents exist^3^. However, poor bioavailability, host toxicities, and the widespread emergence of resistance are driving efforts to identify novel targets and methods for inhibiting fungal growth^4,5^.

The cell wall of fungi mainly consists of glycomannosylated conjugates that create a protective layer against the human immune system^6^. GDP-mannose is a nucleotide sugar metabolite that serves as the substrate for glycosyltransferase enzymes that build the fungal cell wall^7,8^. GDP-mannose transporters are essential for cell survival in fungal species^9,10 11 12^. In addition, the transport of GDP-mannose into the Golgi is crucial for virulence in pathogenic fungi, as well as in parasitic protozoa such as the Trypanosomatidae family^13 14^. However, no antifungal or antiprotozoal drugs currently target GDP-mannose transport, despite these pathways offering promising new directions for drug development.

Nucleotide sugar transporters (NSTs) belong to the Drug and Metabolite Transporter (DMT) superfamily^15^, which in humans forms part of the SLC35 family of solute carriers^16^. Nucleotide sugar transporters operate via a strict antiport mechanism, moving nucleotide sugars into the Golgi in exchange for the free nucleotide monophosphate, which is released back into the cytoplasm^17^. To date, only six structures of DMT family transporters have been reported, recognising a range of metabolites and drugs, including the Plasmodium falciparum chloroquine transporter^18^, the bacterial amino acid transporter YddG^19^, and the human ATP/ADP exchanger^20^. However, only three representative examples of NSTs have been described: the murine CMP-sialic acid transporter^21^, a plant nucleotide transporter that recognises CMP-sialic acid^22^, and Vrg4, the GDP-mannose transporter from *Saccharomyces cerevisiae*^23,24^. These structures reveal that the DMT family shares a conserved 10-transmembrane (TM) architecture, arranged as two inverted topology repeats of five TM helices. Nevertheless, all three NST structures were captured in the Golgi-facing state, which limits insights into how these NSTs recognise ligands from the cytoplasm and undergoes alternating access.

Here, we present the cryo-EM structures of the GDP-mannose transporter, Vrg4, from the pathogenic fungus *Candida albicans*, in complex with nanobodies, in both the Golgi- and cytoplasm-facing conformations. Nanobodies, also known as single-chain antibodies, are increasingly being investigated as potential therapeutic agents across a broad range of medical fields, including cancer, inflammatory conditions, infectious diseases, and neurological disorders^25^. Our functional assays provide proof-of-concept data showing that nanobodies can be used to selectively inhibit the *C. albicans* GDP-mannose transporter Vrg4. Finally, a structure of the cytoplasmic-facing transporter bound to GDP-mannose offers important mechanistic insights into the alternating access transport within the NSTs, with implications for understanding nucleotide sugar transporters within the wider SLC35 family.

## Results

### Identification of inhibitory nanobodies specific for the GDP-mannose transporter from *C. albicans*

**Fig. 1:**
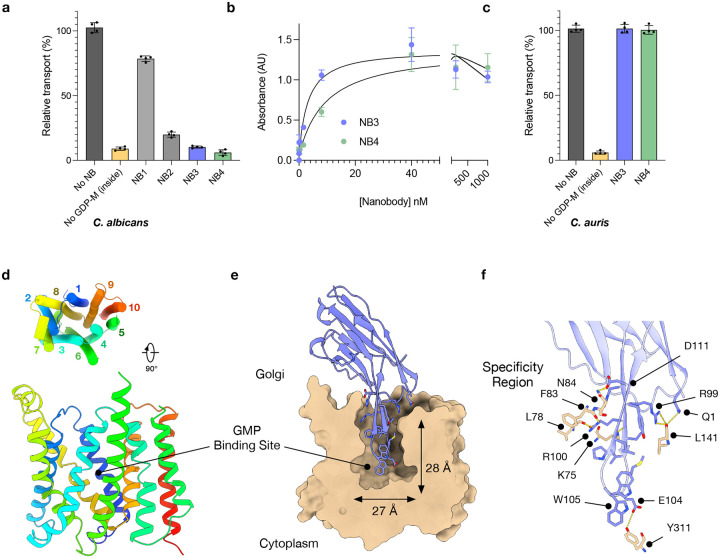
Analysis of inhibitory nanobodies to C. albicans Vrg4. **a**, Transport assay data showing the transport of GMP via C. albicans Vrg4 in the presence of nanobodies (NB) relative to a no NB control. Without GDP-mannose on the inside of the liposome (no GDP-M inside) this transporter cannot function. (n=4 independent experiments performed on different days, the mean is shown, and errors indicate SD). **b**, ELISA data showing normalised absorbance (normalised to the absorbance at 1 μM for each NB) against NB concentration. The calculated KD for NB3 is ~3 nM and for NB4 is ~10 nM. (n=3 independent experiments performed on different days, the mean is shown, and errors indicate SD). **c**, Transport data as in **a** but using C. auris Vrg4 highlighting the specific nature of the NBs. **d**, Cartoon representation of C. albicans Vrg4 in the Golgi facing conformation, with helices coloured from blue to red from the N-terminus. **e**, Surface representation of the cryoEM structure of C. albicans Vrg4 in complex with NB3. The CDR3 loop of NB3 extends into the substrate binding site of the transporter. Shown as sticks are the interacting side chains and the two rotamer positions observed for Trp105. **f**, Zoomed in view of the interactions observed between NB3 (blue) and Vrg4 (wheat).

To identify novel ways to inhibit the GDP-mannose transporter, Vrg4, we immunised llamas with the Vrg4 protein from the pathogenic yeast *Candida albicans*. A library of nanobodies generated from the immunisation was obtained and a subset was screened for the ability to inhibit the activity of CaVrg4 *in vitro* (Extended Data Fig. 1a). We identified four nanobodies that exhibited the ability to block the uptake of ^3^H GMP into liposomes containing CaVrg4, with two nanobodies, NB3 and NB4, showing full inhibition of transport function (Fig. 1a). Nanobodies NB3 and NB4 displayed the highest affinity (K_D_) towards purified CaVrg4, of ~ 3 nM and 10 nM respectively (Fig. 1b & Extended Data Fig. 1b). However, neither NB3 nor NB4 showed any detectable inhibition against Vrg4 from *S. cerevisiae* nor *Candida auris*, which is 84 % similar and 77 % identical to the *C. albicans* homologue (Extended Data Fig. 1d & e & Fig. 1c), highlighting the specific nature of these nanobodies towards *C. albicans*.

### Structural basis for inhibition of *C. albicans* Vrg4.

To understand the mode of inhibition, we next determined the structure of CaVrg4 with both NB3 and NB4. Vrg4 is only 37 kDa and, therefore, a relatively small membrane protein for structure determination using cryo-EM. Nevertheless, we determined the structure of Vrg4 in complex with NB3 at 3.0 Å (Fig. 1d, Table 1 and Extended Data Fig. 2). The structure adopts a similar Golgi-facing state as previously observed in the crystal structure of *S. cerevisiae* Vrg4 (PDB:5OGE)^24^ with an r.m.s.d of 1.47 Å over 288 Ca atoms. The CDR3 loop of NB3 forms an extended beta-hairpin which extends 23 Å into the GDP-mannose binding cavity (Fig. 1e). The loop region of the CDR3 hairpin interacts with regions within the binding site that are important for GDP-mannose and GMP recognition^24,26^. Specifically, Glu104 in the CDR3 loop forms a hydrogen bond with Tyr311 on TM9 (Fig. 1f), which plays an important role in recognising the ribose group in GDP-mannose and is essential for transport activity in *S. cerevisiae* Vrg4^23,24^. Sitting next to Glu104 on the CDR3 loop is Trp105, which is located close to the conserved (F/Y)YNN motif on TM7, responsible for guanine recognition within the NST family. Interestingly, Trp105 adopts two rotamer positions, with one rotamer engaging the guanine pocket and the other packing against Ile134 on TM3. Both rotamers would aid in favourable interactions to secure the CDR3 hairpin in place. The high sequence conservation within the binding sites of GDP-mannose transporters across fungal species raised the question of how specificity is achieved for the *C. albicans* transporter. However, NB3 makes several additional interactions with backbone carbonyl groups in the loop between Tyr76-Phe87 (Fig. 1f); this region of the *C.albicans* protein shows a high level of sequence diversity across Vrg4 sequences (Extended Data Fig. 1d) and hence, while the CDR3 hairpin interacts with highly conserved regions of the transporters, it is the CDR1 and CDR2 regions that drive specificity in this nanobody.

**Fig 2.**
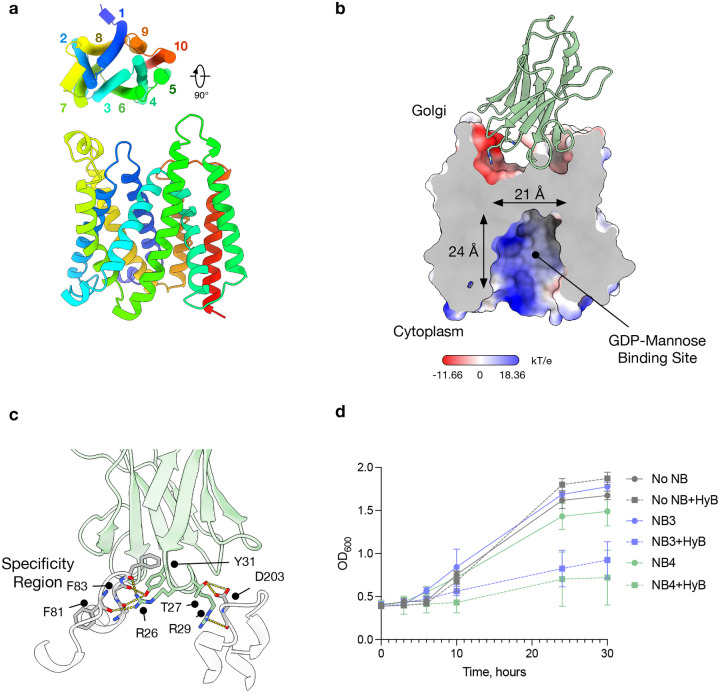
Cytoplasmic facing conformation of Vrg4 with NB4. **a**, Cartoon representation of C. albicans Vrg4 in the cytosolic facing state, with helices coloured from blue to red from the N-terminus. **b**, Electrostatic surface representation of the cryoEM structure of Vrg4 in complex with NB4. Distances shown indicate the size of the binding cavity. **c**, Zoomed in view of the interacting region of NB4 with Vrg4. Shown as sticks are the side chains that contribute to the electrostatic surface. Also shown is the position of Phe81 which sits into a pocket on NB4. **d**, Growth curves of yeast strain NDY5 overexpressing C. albicans Vrg4 and either NB3, NB4 or no NB in the presence (+) and absence of hygromycin B (HyB). In the presence of both NB3 and NB4, the sensitivity of this strain to HyB manifests indicating the inhibition of the function of C. albicans Vrg4. (n=2 independent experiments performed on different days, the mean is shown, and errors indicate SD).

Obtaining the structure of *C. albicans* Vrg4 in complex with NB4 proved more challenging due to a high proportion of uncomplexed particles following the addition of the two proteins prior to grid preparation. However, following enrichment of the sample for the presence of NB4 (see methods), we were able to determine the structure to 3.4 Å (Table 1 & Extended Data Fig. 3). In comparison with the NB3 complex, the NB4-Vrg4 structure adopts the opposing cytoplasmic-facing state of the transporter (Fig. 2a & b). Similar to NB3, the interactions between NB4 and Vrg4 are driven primarily through side chains in CDR1 and CDR2 (Fig. 2c & Extended Data Fig. 1a). In particular, Arg26 and Arg29 in CDR1 make favourable electrostatic interactions with exposed carbonyl groups in loop regions that connect the cytoplasmic ends of the TM helices, while Thr27 hydrogen bonds directly to Asp203, which is unique to *C. albicans*. However, the main driver for specificity originates from a pocket within the nanobody, formed by the interaction between Arg26 and the loop between TM1 and TM2, which accommodates Phe81. Interestingly, CDR3 is pushed to one side of the nanobody scaffold, held away from the main binding interface between Vrg4 and the nanobody. Only two side chains in CDR3 make direct contact with Vrg4. Serine 101 interacts with carbonyl groups on Ile328 and Phe329 on TM9, and Ser99 interacts with Ser79 on TM1. Having established that both NB3 and NB4 interact specifically with *C. albicans* Vrg4, we next wanted to test whether NB3 and NB4 could inhibit the transporter *in vivo*.

For this experiment, we used a strain of *S. cerevisiae* containing a mutant version of the *VRG4* gene (Ala286Asp in the GALNK motif), which confers sensitivity to the antifungal hygromycin B in the yeast^27^. Hygromycin B sensitivity can be alleviated through the presence of a functional copy of Vrg4 and is complemented by the *C. albicans VRG4* gene^27^ (Extended Data Fig. 4a & b). In this complemented yeast strain, we transformed a plasmid containing either NB3 or NB4 with a 5’ secretion signal sequence to target it to the lumen of the Golgi, where our structures indicated both nanobodies interact with CaVrg4. The yeast was grown in minimal media to select for the plasmids, and the expression of the nanobody was induced with galactose. After overnight galactose induction, the yeast culture was diluted, and growth was monitored over 30 hours. The results indicate that in the presence of either NB3 or NB4 hygromycin B causes a slow growth phenotype (Fig. 2d) which is specific for *C. albicans* Vrg4 (Extended Data Fig. 4b). Our growth assays therefore demonstrate that both NB3 and NB4 suppress yeast growth in the presence of hygromycin B and are thus capable of inhibiting CaVrg4 *in vivo*.

### Structural comparison between the Golgi and cytosol-facing states.

**Fig 3.**
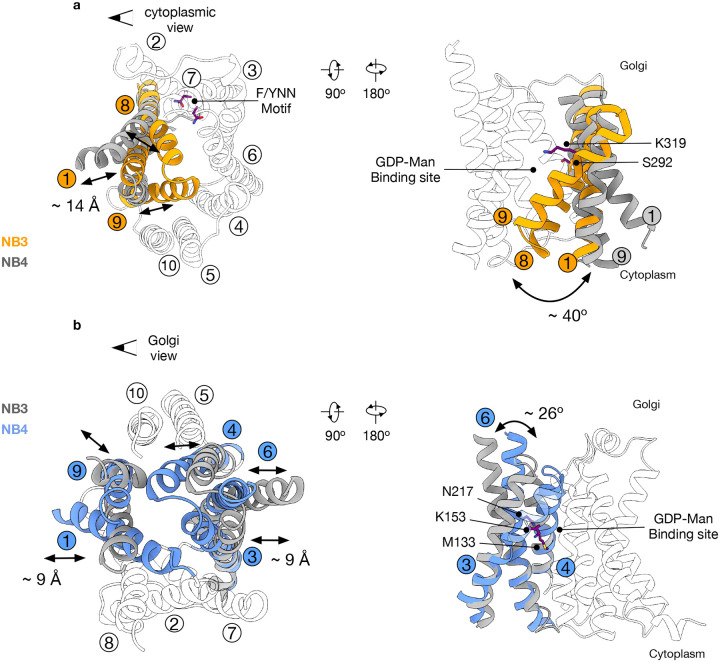
Conformational changes between Golgi and cytoplasmic facing states. **a**, Overlay of Vrg4 in the Golgi open (NB3 bound, orange) and cytoplasmic open (NB4 bound, grey) states highlighting the movement of TMs 1, 8 and 9 (as viewed from the cytoplasm). Residues important for function are indicated as colour sticks and labelled. **b**, Overlay of Vrg4 in the cytoplasm open (NB4 bound, blue) and Golgi open (NB3 bound, grey) states highlighting the movement of TM’s 1, 3, 4, 6 and 9 (as viewed from the Golgi side). Residues important for nucleotide-sugar recognition are indicated as coloured sticks and labelled.

NSTs consist of 10 TM helices that are arranged as two inverted topology repeats of five TMs, such that TMs 1–5 can be superimposed on TMs 6–10 via a 180° rotation in the plane of the membrane^19,24^. Previously, we postulated that transport occurs through the conformational switching of TMs 1, 3 and 4 with TMs 6, 8 and 9, which resulted in the symmetrical pivoting around the central ligand binding site. These structural changes are coordinated with ligand binding through conserved lysines on TMs 4 and 9, which interact with the beta-phosphate and mannose sugar^24^. However, this mechanism was based only on the outward open conformation of *S. cerevisiae* Vrg4 (Golgi lumen-facing) and a symmetrical repeat swapped model of the cytoplasmic-facing state^24^. Our new structures now allow for the direct comparison of the structural rearrangements required for nucleotide-sugar/nucleotide monophosphate exchange. When viewed from the cytoplasmic side of the Golgi membrane we observe that rather than a symmetrical movement of the gating helices, the predominant movement occurs in TMs 1, 8 and 9, with the remaining helices remaining largely static (Fig. 3a). Specifically, to close the cytoplasmic side of the transporter, TMs 1, 8 and 9 move inwards by ~ 14 Å and rotate ~ 40° towards TMs 4, 6 and 7. It is important to note that TM7 contains the conserved nucleotide binding (F/Y)NN^247^ motif, which plays an important role in recognising nucleotide group of the ligand^24^. Thus, the transporter effectively holds the nucleotide in one position whilst the gating helices alternately move to expose the ligand to either the Golgi or cytoplasmic side of the membrane, as discussed in more detail below. Two of the gating helices contain Ser292 (TM8) and Lys319 (TM9), which were previously identified to interact with the ribose group and sugar moiety, respectively^24^. Mutation of Ser292 to alanine reduces transport (Extended Data Fig. 5a), and the equivalent serine on TM8 in *S. cerevisiae* Vrg4 (Ser266) is important for GMP recognition. The lysine on TM9 forms part of the conserved GALNK^319^ motif, which is important for sugar recognition within the NST superfamily^24^. Taken together, the structural rearrangements of TMs 1, 8 and 9 on the cytoplasmic side of the transporter suggest a mechanism whereby the GDP-mannose is recognised and held in place by the largely static F/YNN motif on TM7, whilst the engagement of the ribose and sugar group with TMs 8 and 9 facilitate the structural rearrangements that close the cytoplasmic gate towards the occluded state and luminal release.

In comparison, on the Golgi-facing side of the membrane, the conformational changes are more extensive but smaller (Fig. 3b). To open the Golgi side of the transporter, TMs 1 and 9 move outward by approximately 9 Å, while TM8 remains static. Facing TMs 1 and 9, TMs 3, 4, and 6 also move outward by about 9 Å and rotate away by approximately 26°, pivoting around Met133 (TM3), Lys153 (TM4), and Asn217 (TM6) (Fig. 3b). Mutation of the equivalent lysine or asparagine on TM4 and TM6 to alanine abolishes transport in S. cerevisiae Vrg4^24^. Using our previous repeat-swapped model of the cytoplasmic open state, we proposed that these side chains formed part of the axis of rotation around the ligand during the transport cycle^24^. Our new analysis, utilising the Cryo-EM structures, now shows that while the repeat swap model was qualitatively correct in identifying the helices required for movement, the direct structural comparison reveals important subtleties within the structure during alternating access. An important aspect of this mechanism is that alternating access within Vrg4 is not symmetric, with notable differences in the helix movements on the cytoplasmic and Golgi-facing sides of the transporter. As discussed below, this asymmetry is likely to have significant consequences for ligand recognition within the wider NST family.

### Structural basis for nucleotide sugar recognition from the cytosol.

From the cytoplasmic side of the membrane, Vrg4 must recognise a larger substrate, GDP-mannose, while from the Golgi lumen, the transporter captures GMP for counter-transport to the cytoplasm. Therefore, the asymmetry observed in the gating helices likely plays a significant role in substrate selection. A key consequence of this structural asymmetry is the difference in size of the binding cavity between conformations in the alternating access cycle. In the inward-facing, cytoplasmic open state, the binding cavity is 21 Å wide and 24 Å deep, whereas in the opposite orientation, the cavity measures 27 Å in width and 28 Å in depth. When we overlay the structure of the GDP-mannose bound state of ScVrg4 onto the cytoplasmic open state of CaVrg4, the nucleotide sugar molecule does not fit into the cavity in the same conformation (Extended Data Fig. 5b), implying that the nucleotide sugar must be recognised in a different orientation.

**Fig 4.**
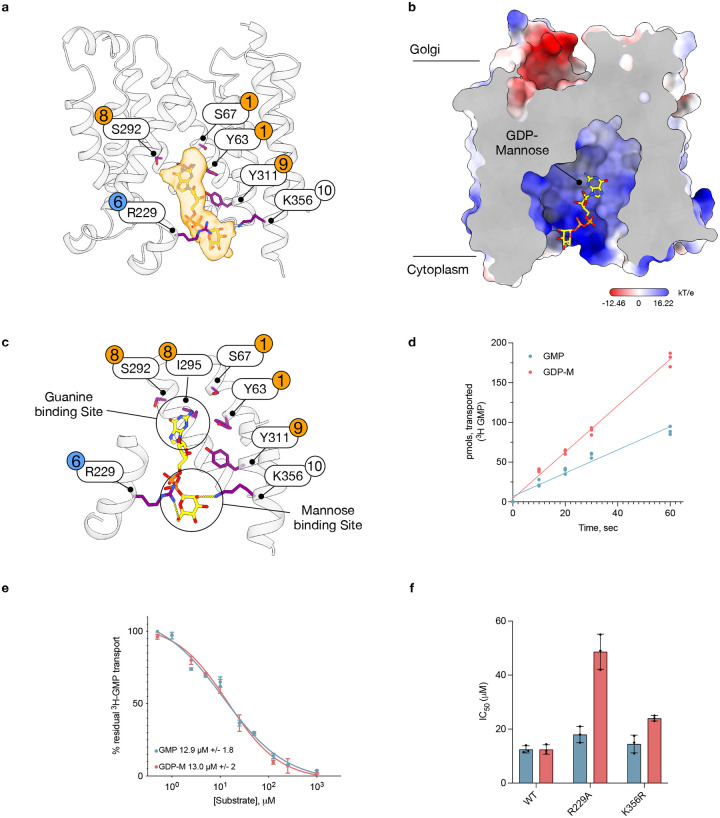
Recognition of GDP mannose in the cytoplasmic facing state. **a**, Cryo-EM density of the GDP-mannose ligand (orange, contoured at a threshold of 0.211). Side chains sitting close (within ~ 4 Å) to the ligand are shown (purple sticks) and labelled with their respective TM helices. **b**, Electrostatic surface representation of Vrg4 bound to GDP-mannose, highlighting the positively charged nature of the binding site. **c**, Zoomed in view of the interactions made with the GDP-mannose. Key side chains are labelled with their respective TM helices. **d**, Initial transport rates of ^3^H GMP uptake into Vrg4 liposomes highlighting the difference when GDP-mannose is used as the antiport substrate versus GMP. (n=3 independent experiments performed on different days). **e**, IC50 values for GMP and GDP mannose for WT Vrg4 for these experiments GDP mannose was used as a counter substrate. (Values shown (mean) are calculated from three independent experiments and errors shown are SD). **f**, Calculated IC50 values for GMP and GDP mannose for WT and mutant forms of Vrg4. (n=3 independent experiments performed on different days, the mean is shown, and errors indicate SD).

Interestingly, in the maps obtained for the NB4-bound structure, we observed density consistent with the endogenous ligand, GDP-mannose (Fig. 4a). The concentration of GDP-mannose in the yeast cytosol is very high (> 10 mM)^28^, which is likely where the ligand was captured during purification. The entrance to the binding site is positively charged, which likely facilitates capture of the GDP-mannose from the cytosol (Fig. 4b). The GDP-mannose sits in a vertical orientation with the guanine group positioned within 4 Å of many side chains that interact with the ligand in the luminal-facing state^24^. These include Tyr63 and Tyr311 (Tyr28 and Tyr281 in S. cerevisiae Vrg4), located near the nucleotide and ribose groups, respectively. Both tyrosines are crucial for function in ScVrg4^24^. Serine 67 on TM 1 is also positioned close to the nucleotide group, along with Serine 292 and 296 (Ser266) on TM 8. Serine 266 in ScVrg4 is vital for nucleotide specificity, which aligns with the modelled position of GDP-mannose in the cytoplasmic-facing state. Interestingly, the two asparagines of the F/YNN motif, Asn246 and Asn247, which play an essential role in nucleotide binding in the Golgi-facing state of ScVrg4, are approximately 8 Å away from the guanine group in the cytoplasmic-facing state (Extended Data Fig. 5c). Notably, the closest interaction between the guanine group and the binding pocket is with Ile295, which, along with the serines discussed above, is also on the gating helix TM 8. Although not immediately apparent, isoleucines have been shown to play important roles in many DNA-binding proteins, forming van der Waals interactions with the nucleobase rings^29^, suggesting a similar interaction may occur within Vrg4 in the Golgi-facing state.

At the opposite end of the ligand, the mannose group interacts with Arg229 on TM6 via the C6 hydroxyl and Lys356 on TM10 via the C2 hydroxyl (Fig. 4c). In the cytoplasmic-facing state, Arg229 forms a salt bridge with Glu164 on TM4. Both TMs 6 and 4 form part of the gating helices in Vrg4 (Fig. 3), suggesting this salt bridge is likely to have functional importance for the transport mechanism. Both ScVrg4 and CaVrg4 display altered kinetics when GMP or GDP-mannose act as the antiport substrate in liposome assays, with GDP-mannose showing faster transport rates and greater overall uptake (Fig. 4d). However, the IC_50_ values for both ligands remain similar, approximately 13 μM (Fig. 4e). If the observed density corresponds to GDP-mannose, then mutation of Arg229 and/or Lys356 might be expected to affect the IC_50_ of GDP-mannose more than that of GMP, considering their interactions with the sugar group. Although a Lys356Ala mutant demonstrated poor transport properties for both ligands (Extended Data 5d), a more conservative mutation to arginine showed transport behaviour akin to the WT protein, as did the Arg229Ala mutation (Extended Data 5). The IC_50_ for GDP-mannose increased to 49 μM (from 13 μM in the WT) in the Arg229Ala mutant, while its impact on GMP was minimal (18 μM versus 13 μM) (Fig. 4e). In comparison, the Lys356Arg mutation had a less significant effect, with the IC_50_ for GDP-mannose rising to 24 μM versus 14 μM for GMP. Therefore, despite the density being suboptimal for precise ligand modelling, the transport assay results support the proposed location of GDP-mannose in the cytoplasmic-facing state.

### Mechanism of nucleotide-sugar transport.

**Fig 5.**
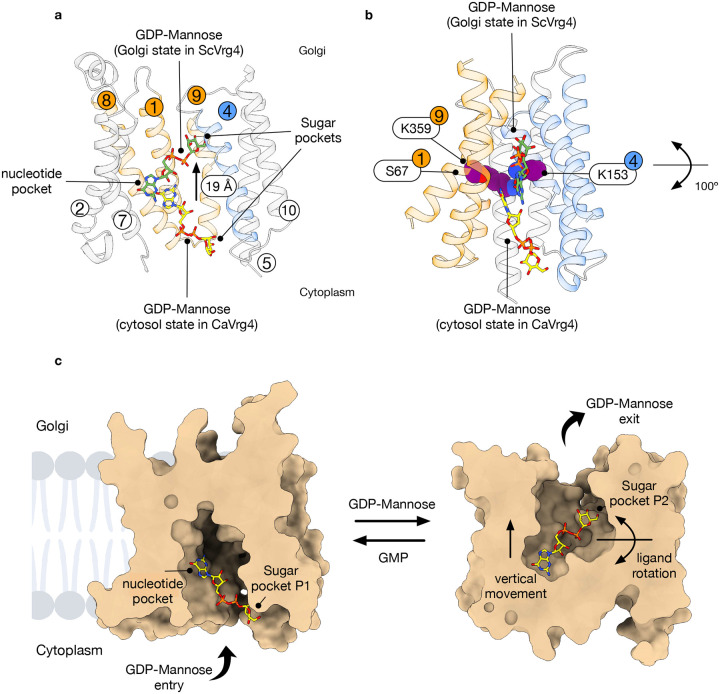
Mechanism of transport in NSTs. **a**, Cartoon representation of CaVrg4 bound to GDP-mannose in the cytoplasmic facing state with the position of the GDP-mannose from the crystal structure of ScVrg4 (PDB: 5OGK) in the Golgi-facing state superimposed. The gating helices are coloured as in Fig. 3. **b**, View of the superimposed structures rotated 90° relative to panel a. Side chains involved in the recognition of GDP-mannose are shown in spheres and labelled with their respective helices. **c**, Model for nucleotide-sugar transport by Vrg4 with the key movements of the GDP-mannose indicated.

Intriguingly, the position of the GDP-mannose in the cytosol-facing state of CaVrg4 differs substantially from the orientation observed in the Golgi-facing state of the ScVrg4 transporter (Fig. 5a). Indeed, the GDP-mannose undergoes an almost 100° movement between the two poses, pivoting around the nucleotide group, with the mannose group moving approximately 19 Å vertically (Fig. 5b). The axis around which the GDP-mannose moves runs through Ser67 on TM1, Lys153 on TM4 and Lys319 on TM9, which are equivalent to Ser32, Lys118 and Lys289 in ScVrg4. These side chains were predicted in our repeat-swapped model of the cytosolic-facing state of Vrg4 to form key hinge regions in the alternating access mechanism, and mutations of these conserved side chains to alanine in ScVrg4 abolish transport^24^. Indeed, Lys319 forms part of the conserved GALNK motif and plays an important role in sugar recognition within the NSTs. Thus, the GDP-mannose undergoes a rotation during transport in the protein that moves the sugar from the cytoplasmic tunnel, where it interacts with Arg229 and Lys356, all the way to the GALNK motif in the Golgi-facing state. Together with the structural comparison discussed above, we therefore propose an updated model for alternating access transport within Vrg4 (Fig. 5c). Starting from the cytoplasmic-facing state, nucleotide sugar ligands are attracted to the transporter via electrostatic interactions, especially at the positively charged binding site. The roles of Arg229 and Lys356 at the entrance to the binding site likely promote interactions with negatively charged molecules. Following initial capture, the GDP-mannose ligand is first positioned through the sugar group, which may provide more specificity than the nucleotide moiety. Considering the higher concentrations of nucleotide phosphates in the cytosol, this may enable the transporter to avoid recognising nucleotide-containing metabolites. Once properly positioned, the GDP-mannose is drawn into the transporter through interactions between the guanine group and the conserved side chains on TMs 1, 8, and 9, which form the nucleotide pocket. The engagement of the nucleotide with TMs 1, 8, and 9 probably triggers these helices to close and shift inward towards TMs 4, 6, and 7. This movement pushes the nucleotide group towards the conserved (F/Y)NN motif on TM 7, which in turn pulls the sugar group down towards the GALNK motif on TM 9 with Tyr 149 on TM 4 acting to coordinate interactions with the ribose group, as observed previously in the crystal structure of the Golgi-facing state^24^. The repositioning of GDP-mannose away from Arg229 and Lys356 allows TMs 3, 4, and 6 to move more freely as the transporter transitions to the Golgi-facing state. The substantial movement of the ligand would require more extensive structural rearrangement in the transporter, which is indeed what we observed in the Golgi-facing side of the protein in our structural comparison (Fig. 3b). This mechanism is atypical for solute carriers, which generally hold the ligand in a fixed position and move around a centrally held ligand to transport it across the membrane^30^. However, it is qualitatively similar to the mechanism recently reported for the structurally homologous SLC35C1 ATP/ADP exchanger in the endoplasmic reticulum, which detailed the vertical repositioning of ATP during the transport cycle^20^. The movement of the GDP-mannose is also consistent with the dynamic binding mode observed for GMP in the crystal structures of the Golgi-facing state of ScVrg4^23^, further supporting the role of ligand dynamics in the transport cycle of the Vrg4 family. The coordinated movement of both the ligand and the gating helices is possibly a conserved feature of the DMT family.

A longstanding question in the NST field has been whether a mechanism exists whereby NSTs facilitate transport direction, ensuring adequate supplies of nucleotide sugars to glycosyltransferases. In Vrg4, we observe faster transport rates for the nucleotide sugar compared to the nucleotide monophosphate (Fig. 4d)^23^. Our proposed mechanism, in which the conjugated sugar group facilitates more efficient repositioning of the nucleotide within the binding site, results in faster transport rates for the nucleotide sugar. The necessity of a ligand to coordinate the gating helices in either the cytoplasmic to Golgi, or Golgi to cytoplasmic facing states also explains the strict antiporter behaviour seen in some members of the SLC35 family^24^.

Finally, the development of new antifungal drugs has recently slowed^3^. Our study demonstrates that blocking nucleotide sugar transport in yeast can halt cell growth, underscoring the potential of nanobodies as therapeutic agents. However, the most likely path forward in drug development will be to identify small-molecule inhibitors. Our structures provide a foundation for structure-guided inhibitor design based on the CDR3 interactions observed in the NB3 complex, revealing a druggable pocket on the Golgi-facing side of the transporter in the NB4 complex.

## Methods

### *Candida albicans* Vrg4 protein expression and purification.

The gene encoding CaVrg4 (UniProt Q5A477) was synthesised as a Gene string from GeneArt and cloned into the pDDGFP-Leu2D vector (Addgene 102334); the first 33 amino acids were excluded. Yeast Strain BJ5460 was transformed and cultivated in synthetic complete medium minus leucine (-leu) with 2% (w/v) D-glucose overnight before being diluted tenfold into -leu containing 2% (v/v) DL-lactate in a 15 L fermentation vessel (Eppendorf BioFlo 415). After 24 hours, expression was induced by adding galactose (final concentration of 1%), and the cells were harvested after a further 20–24 hours. Yeast cells were lysed using high pressure (38 kpsi) and the membrane fraction isolated (at 235,000 g) following a clarification step at 30,000xg. The membranes were washed once in 20 mM HEPES, pH 7.5, 1 M potassium acetate, and finally resuspended in phosphate-buffered saline (PBS). Membranes were solubilised for 1 hour in n-dodecyl-**β**-D-maltoside (DDM, Anatrace) and insoluble material was removed through centrifugation for 1 hour at 235,000xg. 28 mM imidazole was added to the clarified lysate and the His tagged protein was bound to Ni-NTA resin (Thermo) in batch for 4 h at 4 °C. The resin was transferred into a gravity flow column, washed first with 8 column volumes (CV) of solubilisation buffer supplemented with 0.1% (w/v) DDM and 30 mM imidazole and then 25 CV of buffer containing 35 mM imidazole. The protein was eluted with 250 mM imidazole and dialysed overnight (20 mM Tris, pH 7.5, 150 mM NaCl, 0.02% (w/v) DDM) at 4 °C in the presence of Tobacco Etch Virus (TEV) protease. Following a reverse IMAC step the flow through was concentrated using a 50 kDa MWCO spin concentrator (Sartoris) and applied to a Superdex 200 Increase 10/300 GL column (Cytiva) equilibrated in size exclusion buffer (20 mM Tris pH 7.5, 150 mM NaCl and 0.015 (w/v) DDM) for structural determination or in 20 mM Tris pH 7.5, 150 mM NaCl and 0.3 % DM for reconstitution. An Avi-tagged (C terminus) version of the protein was purified as above and biotinylated using BirA ligase for use in Nanobody screening. Mutants were made using site-directed mutagenesis and verified via sequencing, and the protein was produced in the same way as for WT.

### Protein reconstitution into liposomes

CaVrg4 was reconstituted into liposomes using the dilution method into preformed lipid vesicles^31^. Chloroform was removed from the lipids using a rotary evaporator to obtain a thin film. The lipids were washed twice in pentane and then resuspended at 10–20 mg ml^−1^ in lipid buffer (20 mM HEPES, pH 7.5, 100 mM KCl). These lipid vesicles were frozen and thawed twice in liquid nitrogen, then stored at −80°C until use. For reconstitution, the lipids were thawed and then extruded first through a 0.8-μm filter and then through a 0.4-μm filter. The lipid was added to purified Vrg4 in DM (at 0.5 μg μl^−1^ concentration) in batches and at a final lipid: protein ratio of 50:1 and incubated for 1 h at room temperature, then for a further 1 h on ice. After this time, the protein–lipid mix was diluted rapidly into 65 mL of assay buffer (20 mM HEPES, pH 7.5, 50 mM KCl, 2 mM MgSO4) and the proteoliposomes were collected through centrifugation at >200,000xg for 2 h. To remove trace detergent, the proteoliposomes were dialysed overnight against a large volume (3 L) of assay buffer. After dialysis, the proteoliposomes were collected and resuspended in assay buffer to a final protein concentration of 0.5 μg μl^−1^, and then subjected to two rounds of freeze–thawing in liquid nitrogen before storage at −80 °C. The amount of protein reconstituted into the lipids was quantified by SDS–PAGE and densitometry. For immunisation, the lipid mix consisted of POPE: POPG at a 3:1 ratio, and the final lipid: protein ratio was 30:1. The final buffer was PBS. For all the assays, the lipid mix consisted of POPE:POPG:*Ecoli* Polar lipid Extract:EggPC in a 27:7:9:1 ratio.

### Nanobody selection and purification

To identify CaVrg4 specific nanobodies a library was raised through immunisation of a llama using reconstituted protein (reconstituted in POPE:POPG lipids at 3:1 ratio) as detailed in (Pardon 2014). Reconstituted material was injected intramuscularly using Gerbu LQ#3000 as the adjuvant. Immunisations and handling of the llama were performed under the authority of the project license PPL 70/8108. A 150 ml blood sample was collected, and peripheral blood mononuclear cells were prepared using Ficoll-Paque PLUS. Total RNA was extracted using TRIzolTM, and VHH cDNAs were generated by reverse transcription-PCR. Two rounds of nested PCR amplified the pool of VHH encoding sequences: firstly with ‘CALL_001’ and ‘CALL_002’, followed by ‘VHH_For’ and ‘VHH_Rev_IgG2’ and ‘VHH_Rev_IgG3’, and cloned into the SfiI sites of the phagemid vector pADL-23c. In this vector, the VHH encoding sequence is preceded by a pelB leader sequence and has a C-terminal His-Myc Tag. Electro-competent TG1 cells were transformed with the recombinant pADL-23c vector, resulting in a VHH library of about 2 × 10^8^ independent transformants. The resulting TG1 library stock was then infected with M13K07 helper phage to obtain a library of VHH-presenting phages. Phages displaying VHHs specific for CaVrg4 were enriched after five rounds of biopanning using 50 nM of biotinylated protein and capturing with Dynabeads MyOne Straptavidin T1 (Thermo Fisher). After the fifth round of panning, 95 individual phagemid clones were picked, VHH displaying phages were recovered by infection with M13K07 helper phage and tested for binding for specific Vrg4 binding by ELISA. ELISA-positive clones were sequenced, and unique nanobodies were identified. Unique nanobodies that appeared more than three times within the sequenced ELISA-positive hits were phylogenetically analysed, and from this analysis, 10 nanobodies, which were most distantly related, were purified on a 50 mL scale using nickel resin. Four nanobodies that expressed to the highest level in this vector and could interact with CaVrg4 via a nickel pulldown were subsequently subcloned into the pSBinit vector (Addgene 110100) and purified. The binding properties were further analysed via ELISA, and the impact of these nanobodies on the transport efficiency of CaVrg4 was examined.

### Cryo-EM sample preparation and data acquisition

CaVrg4 post gel filtration was incubated with 1.5 molar excess of NB3 for 4 hours on ice at a final Vrg4 concentration of 6 mg/ml. For the complex with NB4, this strategy results in a high proportion of uncomplexed particles; therefore, the Vrg4-NB complex was enriched by binding it to nickel resin. In brief, Vrg4 at 0.5 mg/mL was incubated with a 3-fold molar excess of NB4 for 2 hours on ice and then bound to nickel resin for 1 hour. After extensive washing to remove uncomplexed Vrg4, the complex was eluted and dialysed against DDM size exclusion buffer.

Four microliters of CaVrg4-nb3 (4.4 mg/ml) or CaVrg4-nb4 (4.0 mg/ml) were adsorbed onto glow-discharged holey carbon-coated grids (Quantifoil 300 mesh, Au R1.2/1.3) for 10 s. Grids were blotted for 2 s at 10 °C, 100% humidity and frozen in liquid ethane using a Vitrobot Mark IV (Thermo Fisher Scientific). Movies were collected in counted mode, in Electron Event Representation (EER) format, on a CFEG-equipped Titan Krios G4 (Thermo Fisher Scientific) operating at 300 kV with a Selectris X imaging filter (Thermo Fisher Scientific) and slit width of 10 eV, at 165,000x magnification on a Falcon 4i direct detection camera (Thermo Fisher Scientific), corresponding to a calibrated pixel size of 0.732 Å. Movies were collected at a total dose of 55.0 or 53.7 e^−^/A^2^ (Table 1), fractionated to ~ 1.0 e^−^ / Å^2^ per fraction for motion correction.

### Cryo-EM data processing

Patched motion correction, CTF parameter estimation, particle picking, extraction, and initial 2D classification were performed in SIMPLE 3.0^32^. All downstream processing was carried out in cryoSPARC^33^ or RELION 3.1^34^, using the csparc2star.py script within UCSF pyem^35^ to convert between formats. Global resolution was estimated from gold-standard Fourier shell correlations (FSCs) using the 0.143 criterion and local resolution estimation was calculated within cryoSPARC.

The cryo-EM processing workflow for CaVrg4-nb3 is outlined in Extended Data Fig. 2. Briefly, particles (2,647,181) were subjected to two rounds of reference-free 2D classification in cryoSPARC (1^st^ classification using 300 classes, 2^nd^ classification using 200 classes) using first a 160 Å then 140 Å soft circular mask. Selected particles (638,060) were subjected to multi-class *ab initio* reconstruction using a maximum resolution cutoff of 6 Å, generating four volumes. Particles (348,612) from the two most populated and structured classes were selected and non-uniform refined against one of their corresponding volumes lowpass-filtered to 15 Å, generating a 3.3 Å map. Bayesian polishing in RELION further improved map quality to 3.1 Å after non-uniform refinement. Local CTF refinement and global CTF refinement (fitting beam tilt and trefoil) followed by non-uniform refinement yielded a 3.01 Å volume. The map was globally sharpened within cryoSPARC using the B-factor calculated from the Guinier plot.

The cryo-EM processing workflow for CaVrg4-nb4 is outlined in Extended Data Fig. 3. Briefly, particles (8,692,632) underwent two rounds of reference-free 2D classification in cryoSPARC (1^st^ classification using 300 classes, 2^nd^ classification using 200 classes) using first a 160 Å and then 140 Å soft circular mask. Selected particles (1,074,584) were subjected to multi-class *ab initio* reconstruction with a maximum resolution cutoff of 6 Å, resulting in four volumes. Particles (325,091) from the most populated and structured class were selected and non-uniformly refined against one of their corresponding volumes lowpass-filtered to 15 Å, generating a 3.3 Å map. Bayesian polishing in RELION, followed by an additional round of 2D classification (k=100, with a 140 Å soft circular mask), led to the selection of 208,420 pruned particles, which were non-uniformly refined to produce a 3.2 Å volume. Local CTF refinement (fitting per-particle defocus) followed by non-uniform refinement resulted in a marginally improved 3.2 Å volume. To enhance density for GDP-mannose, alignment-free 3D classification was performed in RELION (k=4, no resolution filter, regularization parameter T=40) using a soft mask focused on the transporter cavity. This produced one class containing 51,142 particles, which could be non-uniform refined to 3.4 Å resulting in improved density for GDP-mannose. Maps were globally sharpened within cryoSPARC using the B-factor calculated from the Guinier plot or sharpened using deepEMhancer^36^.

### Model building and refinement

A model of CaVrg4, based on the ScVrg4 structure, and a nanobody structure, based on the NB from 7BC6, were docked into the globally sharpened map, adjusted where necessary by manual building using Coot v. 0.9^37^ and real-space refinement in PHENIX v. 1.21.2–5419^38^ using secondary structure, rotamer, and Ramachandran restraints. Ligand restraints were generated using Grade2^39^. The final models were validated using MolProbity^40^ within PHENIX. Figures were prepared using UCSF ChimeraX v.1.9^41^.

### Transport assays

To analyse the effect of the nanobodies on Vrg4 activity *in vitro*, proteoliposomes containing CaVrg4 (or Vrg4 from *Saccharomyces cerevisiae*, or *Candida auris* as specificity controls) were incubated with 20 molar excess of nanobody. After an hour, the sample was diluted 20 times (to 500 μl) and 0.4 mM GDP-mannose was added. The liposomes were subjected to four rounds of freeze-thaw in liquid nitrogen and extruded through a 0.4-μm membrane. The liposomes were harvested through ultracentrifugation (120,000 g for 25 mins at 18 °C) and resuspended in assay buffer. The equivalent of 2 μg of protein was added to 50 μl of assay buffer containing 0.5 μM ^3^H-GMP (Hartmann analytic), which initiated the transport. All assays were performed at 30 °C. The uptake of radiolabeled substrate was stopped at the desired time (5 minutes) by rapidly filtering onto 0.22-μm filters, which were then washed with 2 × 2 mL of cold water. The amount of GMP transported inside the liposomes was calculated by scintillation counting in Ultima Gold (Revvity).

### Hygromycin B-based *in vivo* viability assay.

For *in vivo* functional characterization of the identified nanobodies the yeast NDY5 strain (MAT ura3-52a, leu2-211, vrg4-2) was transformed with an episomal vector containing either VRG4 from *C. albicans* or *S. cerevisiae*, under the control of the ADH promoter (yEP181, leucine marker) and also a yEP vector containing the NB under the control of the GAL promoter (yEP195, ura marker). An overnight culture was grown in minus -Leu-Ura medium containing 2% glucose and then diluted 1:10 into medium containing 2% lactate. After 9 hours of growth 2% galactose was added, and the cultures were left overnight. These cultures were diluted into fresh -Leu-Ura media with 2 % galactose at an OD of 0.4 and split into two flasks, to one a final concentration of 0.085 mg/ml hygromycin B was added. The cultures were grown at 30 °C with shaking, and growth was monitored over the next 30 hours by measuring the OD. Measurements were performed in duplicate, and the whole experiment was repeated to obtain the means shown in the figures.

### ELISA to determine K_D_.

The purified nanobodies from the pSBinit vector contain a C terminal Myc-His tag. The myc epitope was used to bind a dilution series of each nanobody to MaxiSorp plates containing protein A and anti-C-Myc antibody. Protein A (5 mg/ml) was diluted 1:10,000 in PBS, and 100 μL was added per well. The plate was then left overnight. After washing and blocking for 30 minutes in (0.5% BSA in PBS), 100 μl of 1:2000 anti-c-myc antibody was added in PBS+0.03% DDM and left for 45 minutes. The plates were washed, and then a dilution series of the nanobody of interest was added and left for 30 minutes. Following 3 × 200 μl washes, 100 μl of 50 nM biotinylated CaVrg4 (or *Cauris* Vrg4 as a control) was added for 40 minutes. The plates were washed before adding 100 μl of 1:5000 diluted streptavidin-peroxidase polymer, which was left for 40 minutes. After washing, the plates were developed using TMB (Merck T2885) and the subsequent absorbance was read at 650 nm. The background was subtracted (absorbance with no binder attached to the plate), and the absorbance of the highest binder concentration was set to 1. The KD was calculated in GraphPad using the “one site – total binding” equation. The experiment was repeated in triplicate to obtain a mean and standard error.

## Supplementary Material

Supplementary Files

This is a list of supplementary les associated with this preprint. Click to download.

- ExtendedDataFigure15.pdf

## Figures and Tables

**Table 1.** Cryo-EM data collection, refinement, and validation statistics

	CaVrg4-NB3 (EMDB-54523) (PDB 9S35)	CaVrg4-NB4-GDP-mannose (EMDB-54524) (PDB 9S36)
**Data collection and processing**		
Magnification	165,000	165,000
Voltage (kV)	300	300
Electron exposure (e–/Å^2^)	55.0	53.7
Defocus range (μm)	−2.0 to −0.5	−2.0 to −0.8
Pixel size (Å)	0.732	0.732
Symmetry imposed	C1	C1
Initial particle images (no.)	7,870,980	8,921,772
Final particle images (no.)	348,612	51,142
Map resolution (Å)	3.0	3.4
FSC threshold	0.143	0.143
**Refinement**		
Initial model used (PDB code)	ScVrg4 (5OGK)	
Model composition in the asymmetric unit		
Non-hydrogen atoms	3361	3455
Protein residues	428	441
Ligands	0	1
Average *B* factors (Å^2^)		
Protein	26.53	52.70
Ligand		95.50
R.m.s. deviations		
Bond lengths (Å)	0.004	0.002
Bond angles (°)	0.534	0.463
Validation		
MolProbity score	1.67	1.39
Clashscore	5.0	4.0
Poor rotamers (%)	2.2	0.50

## Data Availability

Atomic coordinates for CaVrg4 have been deposited in the Protein Data Bank under accession codes 9S35 (NB3); 9S36 (NB4/GDP mannose). The cryo-EM maps have been deposited in the Electron Microscopy Data Bank (EMDB) under accession codes EMD-54523 (NB3); EMD-54524 (NB4/GDP-mannose).

## References

[1] Kauffman C.A. Fungal infections. Proc Am Thorac Soc 3, 35–40 (2006). 16493149 10.1513/pats.200510-110JH

[2] Morad H.O.J. Pre-clinical Imaging of Invasive Candidiasis Using ImmunoPET/MR. Front Microbiol 9, 1996 (2018). 30190717 10.3389/fmicb.2018.01996 PMC6115526

[3] Puumala E., Fallah S., Robbins N. & Cowen L.E. Advancements and challenges in antifungal therapeutic development. Clin Microbiol Rev 37, e0014223 (2024). 38294218 10.1128/cmr.00142-23 PMC10938895

[4] Perfect J.R. The antifungal pipeline: a reality check. Nat Rev Drug Discov 16, 603–616 (2017). 28496146 10.1038/nrd.2017.46 PMC5760994

[5] Berkow E.L. & Lockhart S.R. Fluconazole resistance in Candida species: a current perspective. Infect Drug Resist 10, 237–245 (2017). 28814889 10.2147/IDR.S118892 PMC5546770

[6] Dakalbab S. Uniqueness of Candida auris cell wall in morphogenesis, virulence, resistance, and immune evasion. Microbiol Res 286, 127797 (2024). 38851008 10.1016/j.micres.2024.127797

[7] Hirschberg C.B. My journey in the discovery of nucleotide sugar transporters of the Golgi apparatus. J Biol Chem 293, 12653–12662 (2018). 30120148 10.1074/jbc.X118.004819 PMC6102126

[8] Hirschberg C.B., Robbins P.W. & Abeijon C. Transporters of nucleotide sugars, ATP, and nucleotide sulfate in the endoplasmic reticulum and Golgi apparatus. Annu Rev Biochem 67, 49–69 (1998). 9759482 10.1146/annurev.biochem.67.1.49

[9] Jackson-Hayes L. Two GDP-mannose transporters contribute to hyphal form and cell wall integrity in Aspergillus nidulans. Microbiology 154, 2037–47 (2008). 18599832 10.1099/mic.0.2008/017483-0

[10] Nishikawa A., Poster J.B., Jigami Y. & Dean N. Molecular and phenotypic analysis of CaVRG4, encoding an essential Golgi apparatus GDP-mannose transporter. J Bacteriol 184, 29–42 (2002). 11741841 10.1128/JB.184.1.29-42.2002 PMC134776

[11] Wang Z.A. Cryptococcus neoformans dual GDP-mannose transporters and their role in biology and virulence. Eukaryot Cell 13, 832–42 (2014). 24747214 10.1128/EC.00054-14 PMC4054277

[12] Kadry A.A., El-Ganiny A.M., Mosbah R.A. & Kaminskyj S.G.W. Deletion of Aspergillus nidulans GDP-mannose transporters affects hyphal morphometry, cell wall architecture, spore surface character, cell adhesion, and biofilm formation. Med Mycol 56, 621–630 (2018). 29420778 10.1093/mmy/myx082

[13] Guo H. Genetic metabolic complementation establishes a requirement for GDP-fucose in Leishmania. J Biol Chem 292, 10696–10708 (2017). 28465349 10.1074/jbc.M117.778480 PMC5481574

[14] Descoteaux A., Luo Y., Turco S.J. & Beverley S.M. A specialized pathway affecting virulence glycoconjugates of Leishmania. Science 269, 1869–72 (1995). 7569927 10.1126/science.7569927

[15] Jack D.L., Yang N.M. & Saier M.H. Jr. The drug/metabolite transporter superfamily. Eur J Biochem 268, 3620–39 (2001). 11432728 10.1046/j.1432-1327.2001.02265.x

[16] Ishida N. & Kawakita M. Molecular physiology and pathology of the nucleotide sugar transporter family (SLC35). Pflgers Archiv European Journal of Physiology 447, 768–775 (2004). 12759756 10.1007/s00424-003-1093-0

[17] Capasso J. & Hirschberg C. Mechanisms of glycosylation and sulfation in the Golgi apparatus: evidence for nucleotide sugar/nucleoside monophosphate and nucleotide sulfate/nucleoside monophosphate antiports in the Golgi apparatus membrane. Proc Natl Acad Sci U S A 81, 7051–7055 (1984). 6095266 10.1073/pnas.81.22.7051 PMC392074

[18] Kim J. Structure and drug resistance of the Plasmodium falciparum transporter PfCRT. Nature 576, 315–320 (2019). 31776516 10.1038/s41586-019-1795-x PMC6911266

[19] Tsuchiya H. Structural basis for amino acid export by DMT superfamily transporter YddG. Nature 534, 417–20 (2016). 27281193 10.1038/nature17991

[20] Gulati A. Stepwise ATP translocation into the endoplasmic reticulum by human SLC35B1. Nature (2025). 10.1038/s41586-025-09069-w PMC12267056 40399679

[21] Ahuja S. & Whorton M.R. Structural basis for mammalian nucleotide sugar transport. Elife 8(2019). 10.7554/eLife.45221 PMC6508934 30985278

[22] Nji E., Gulati A., Qureshi A.A., Coincon M. & Drew D. Structural basis for the delivery of activated sialic acid into Golgi for sialyation. Nat Struct Mol Biol 26, 415–423 (2019). 31133698 10.1038/s41594-019-0225-y

[23] Parker J.L., Corey R.A., Stansfeld P.J. & Newstead S. Structural basis for substrate specificity and regulation of nucleotide sugar transporters in the lipid bilayer. Nat Commun 10, 4657 (2019). 31604945 10.1038/s41467-019-12673-w PMC6789118

[24] Parker J.L. & Newstead S. Structural basis of nucleotide sugar transport across the Golgi membrane. Nature 551, 521–524 (2017). 29143814 10.1038/nature24464 PMC5701743

[25] Steeland S., Vandenbroucke R.E. & Libert C. Nanobodies as therapeutics: big opportunities for small antibodies. Drug Discov Today 21, 1076–113 (2016). 27080147 10.1016/j.drudis.2016.04.003

[26] Gao X., Nishikawa A. & Dean N. Identification of a conserved motif in the yeast golgi GDP-mannose transporter required for binding to nucleotide sugar. J. Biol. Chem. 276, 4424–4432 (2001). 11067855 10.1074/jbc.M009114200

[27] Poster J.B. & Dean N. The yeast VRG4 gene is required for normal Golgi functions and defines a new family of related genes. J Biol Chem 271, 3837–45 (1996). 8632002 10.1074/jbc.271.7.3837

[28] Mattila P., Räbinä J., Hortling S., Helin J. & Renkonen R. Functional expression of Escherichia coli enzymes synthesizing GDP-L-fucose from inherent GDP-D-mannose in Saccharomyces cerevisiae. Glycobiology 10, 1041–1047 (2000). 11030750 10.1093/glycob/10.10.1041

[29] Jakubec D., Laskowski R.A. & Vondrasek J. Sequence-Specific Recognition of DNA by Proteins: Binding Motifs Discovered Using a Novel Statistical/Computational Analysis. PLoS One 11, e0158704 (2016). 27384774 10.1371/journal.pone.0158704 PMC4934765

[30] Drew D. & Boudker O. Shared Molecular Mechanisms of Membrane Transporters. Annu Rev Biochem 85, 543–72 (2016). 27023848 10.1146/annurev-biochem-060815-014520

[31] Parker J.L., Mindell J.A. & Newstead S. Thermodynamic evidence for a dual transport mechanism in a POT peptide transporter. Elife 3(2014). 10.7554/eLife.04273 PMC4271188 25457052

[32] Caesar J. SIMPLE 3.0. Stream single-particle cryo-EM analysis in real time. J Struct Biol X 4, 100040 (2020). 33294840 10.1016/j.yjsbx.2020.100040 PMC7695977

[33] Punjani A., Zhang H. & Fleet D.J. Non-uniform refinement: adaptive regularization improves single-particle cryo-EM reconstruction. Nat Methods 17, 1214–1221 (2020). 33257830 10.1038/s41592-020-00990-8

[34] Zivanov J., Nakane T. & Scheres S.H.W. A Bayesian approach to beam-induced motion correction in cryo-EM single-particle analysis. IUCrJ 6, 5–17 (2019). 10.1107/S205225251801463X PMC6327179 30713699

[35] Asarnow D., Palovcak E. & Cheng Y. UCSF pyem v0.5, (2019).

[36] Sanchez-Garcia R. DeepEMhancer: a deep learning solution for cryo-EM volume post-processing. Commun Biol 4, 874 (2021). 34267316 10.1038/s42003-021-02399-1 PMC8282847

[37] Brown A. Tools for macromolecular model building and refinement into electron cryo-microscopy reconstructions. Acta Crystallogr D Biol Crystallogr 71, 136–53 (2015). 25615868 10.1107/S1399004714021683 PMC4304694

[38] Afonine P.V. Real-space refinement in PHENIX for cryo-EM and crystallography. Acta Crystallogr D Struct Biol 74, 531–544 (2018). 29872004 10.1107/S2059798318006551 PMC6096492

[39] Smart O.S. Grade2 1.5.0 edn (Global Phasing Ltd., Cambridge, United Kingdom, 2021).

[40] Prisant M.G., Williams C.J., Chen V.B., Richardson J.S. & Richardson D.C. New tools in MolProbity validation: CaBLAM for CryoEM backbone, UnDowser to rethink “waters,” and NGL Viewer to recapture online 3D graphics. Protein Sci 29, 315–329 (2020). 31724275 10.1002/pro.3786 PMC6933861

[41] Pettersen E.F. UCSF ChimeraX: Structure visualization for researchers, educators, and developers. Protein Sci 30, 70–82 (2021). 32881101 10.1002/pro.3943 PMC7737788

